# Phase Stability and Electrochemical Performance of La-Site-Doped Li_6_La_3_Zr_0.5_Nb_0.5_Ta_0.5_Hf_0.5_O_12_ High-Entropy Garnets

**DOI:** 10.1021/acs.inorgchem.5c05633

**Published:** 2026-01-15

**Authors:** Chang Li, Nava Raj Giri, Zhihao Jin, Yan Chen, Zhezhen Fu

**Affiliations:** † Mechanical Engineering, School of Science, Engineering and Technology, The Pennsylvania State University, Harrisburg, Middletown, Pennsylvania 17057, United States; ‡ Neutron Scattering Division, 6146 Oak Ridge National Laboratory, Oak Ridge, Tennessee 37831, United States

## Abstract

We investigate La-site substitution in the high-entropy garnet Li_6_La_3_Zr_0.5_Nb_0.5_Ta_0.5_Hf_0.5_O_12_ (LLZNTH) using Ba^2+^, Sr^2+^, and Sm^3+^ to elucidate how dopant governs phase stability, Li-site distribution, and electrochemical behavior. X-ray diffraction shows that Sr^2+^ is incorporated homogeneously into the garnet lattice, whereas the larger Ba^2+^ and smaller Sm^3+^ ions partially exceed the structural tolerance, generating secondary phases. Nevertheless, the Sm-doped composition (*x* = 0.05) exhibits the highest room-temperature ionic conductivity (2.7 × 10^–4^ S cm^–1^). Neutron powder diffraction reveals that Sm substitution drives a redistribution of Li^+^ from the tetrahedral 24 d sites into the higher-mobility 96 h positions, enhancing the connectivity of the three-dimensional Li-ion migration network. A Sm-doping series (*x* = 0.01–0.05) further shows that only sufficiently high Sm levels induce this redistribution, whereas lower concentrations retain Li arrangements similar to the undoped garnet. Critical current density measurements demonstrate that La-site dopants also influence interfacial stability against Li metal, underscoring a trade-off between bulk transport enhancement and mechanical robustness. Collectively, these findings reveal that in high-entropy garnets improved ionic conductivity can originate not only from phase-pure structures but also from targeted modification of the Li sublattice, even when accompanied by secondary phases, offering a compositional design principle for garnet electrolytes.

## Graphical abstract

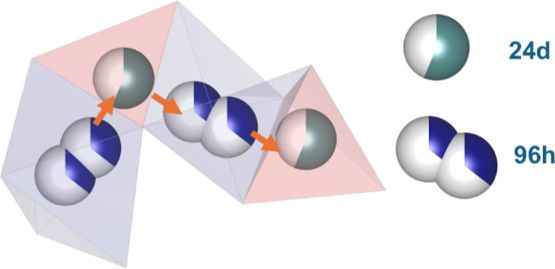

## 1 Introduction

Li-ion batteries have become indispensable for applications ranging from consumer electronics to electric vehicles and grid-scale energy storage.^1,2^ However, their further development is constrained by limitations in energy density and safety, primarily associated with flammable liquid electrolytes and the use of graphite anodes.^3–7^ Solid-state batteries (SSBs), which employ nonflammable solid-state electrolytes (SSEs) together with high-capacity Li metal anodes, offer a promising pathway to overcome these limitations by enabling both higher energy density and improved safety.^4,5,7–10^

Among the various classes of SSEs, Li-garnet oxides stand out due to their high ionic conductivity, wide electrochemical stability window, good stability under ambient conditions, and mechanical robustness. The most studied garnet, Li_7_La_3_Zr_2_O_12_ (LLZO), exhibits a cubic phase with the *Ia*3̅*d* space group and an ionic conductivity on the order of 10^–4^ S cm^-1^.^9–14^ Extensive research has demonstrated that aliovalent doping is essential for stabilizing the cubic phase and enhancing the ionic conductivity. For instance, Al substitution at the Li site stabilizes the cubic phase through controlled Li-vacancy formation,^15^ while Ta and Nb substitution at the Zr site also effectively promote cubic stability and high conductivity.^16–22^ To date, most reports focus on single-element doping strategies, and the conductivity of garnet electrolytes typically remains in the range of 1 × 10^–4^ S cm^–1^.

Inspired by the concept of high-entropy ceramics (HECs), which utilize multiple cations to induce configurational entropy stabilization and unique property tuning,^21,23–25^ high-entropy Li-garnets have recently attracted increasing attention.^26–31^ The incorporation of multiple cations at Zr or La sites introduces local lattice distortions, cation disorder, and site-energy overlaps, which can reduce migration barriers and facilitate Li-ion transport.^21,27,32–36^ However, further optimization is still needed to balance the phase stability, densification, and Li-ion conductivity. La-site doping in Li-garnet electrolytes has also been reported to play an important role, as it can modify lattice parameters and influence ionic conductivity;^34–37^ however, its impact on Li occupancy remains unclear, and its role within high-entropy garnet systems is still not well understood.

In this study, we explore La-site doping with three different cations, Ba, Sr, and Sm, in the LLZNTH framework, extending our previously reported high-entropy Li-garnet system derived from Zr-site multicomponent substitution.^26,38^ The rationale for this selection is 3-fold. First, Ba^2+^ and Sr^2+^, with their larger ionic radii compared to La^3+^, are expected to introduce significant local lattice distortions and modify bottleneck sizes for Li-ion migration.^35,36^ Second, Sm^3+^ has a comparable oxidation state to La^3+^ but a smaller ionic radius, potentially tuning lattice disorder while preserving charge balance.^39^ Third, these substitutions probe the solubility limit and phase formation tendencies of La-site doping in high-entropy garnet, offering insights into how dopant chemistry affects both the structural stability and ionic transport. The design principles derived from these dopants are expected to be broadly applicable to other high-entropy garnet and multicomponent oxide systems. By systematically examining Ba-, Sr-, and Sm-substituted LLZNTH through X-ray diffraction (XRD), scanning electron microscopy with energy-dispersive X-ray spectroscopy (SEM/EDS), electrochemical impedance spectroscopy (EIS), and neutron powder diffraction (NPD), we aim to establish clear correlations among dopant type, phase evolution, Li-site occupancy, and ionic conductivity. This work not only elucidates the role of La-site dopants in high-entropy garnets but also provides guidelines for the rational design of next-generation garnet-type solid electrolytes.

## 2 Results and Discussion

Figure 1 shows the room-temperature XRD patterns of the parent LLZNTH composition (Li_6_La_3_Zr_0.5_Nb_0.5_Ta_0.5_Hf_0.5_O_12_) together with samples doped at the La site with Ba, Sr, and Sm (*x* = 0.05). Table 1 lists the corresponding phase assignments, relative densities, and ionic conductivities. All of the samples retained the cubic garnet framework (space group *Ia*3̅*d*, PDF 80-0457). Still, differences in phase purity and secondary phase formation can be seen depending on the size and charge of the substituent cations. This indicates that phase stability is closely governed by the dopant’s ionic radius and oxidation state. Sr^2+^, with moderate size mismatch and charge imbalance compensated by Li vacancies, stabilizes a single-phase structure, whereas the larger Ba^2+^ and smaller Sm^3+^ induce local strain that promotes secondary phase formation.

**Figure 1:**
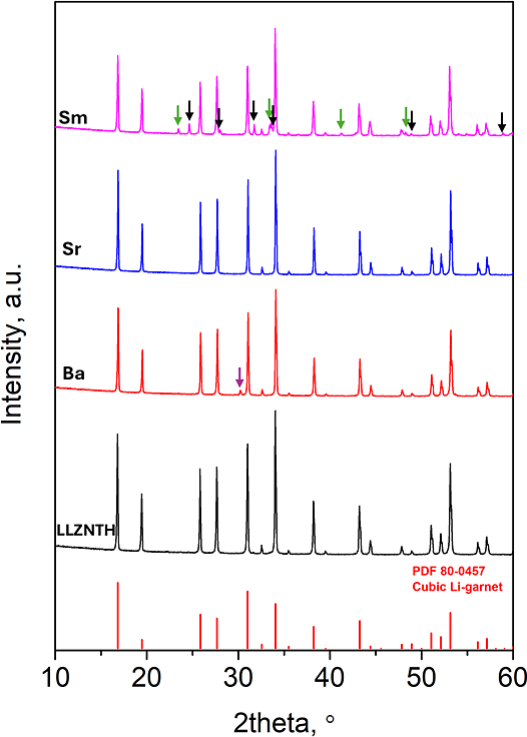
XRD patterns of the samples: undoped LLZNTH, 0.05Ba-doped, 0.05Sr-doped, and 0.05Sm-doped. The purple arrow on the Ba pattern represents Ba_2_LaZrO_5.5_ (PDF 48-0334). Green arrows on the Sm pattern represent LaAlO_3_ (PDF 31-0022), and black arrows on the Sm pattern represent La_2_Li_0.5_Al_0.5_O_4_ (PDF 40-1167).

**Table 1:** Compositions, Relative Densities, Phase Information, and Ionic Conductivities of Undoped and La-Site-Doped LLZNTH Samples

sample	composition	relative density (%)	phase(s)	ionic conductivity (10^–4^ S cm^–1^)	remarks
LLZNTH	Li_6_La_3_Zr_0.5_Nb_0.5_Ta_0.5_Hf_0.5_O_12_	∼89	single cubic garnet	1.7	fine grains, porous
0.05Ba	Li_6_La_2.95_Ba_0.05_Zr_0.5_Nb_0.5_Ta_0.5_Hf_0.5_O_12_	∼91	cubic garnet, Ba_2_LaZrO_5.5_	1.9	grain growth, segregation
0.05Sr	Li_6_La_2.95_Sr_0.05_Zr_0.5_Nb_0.5_Ta_0.5_Hf_0.5_O_12_	∼95	single cubic garnet	2.3	single phase, uniform
0.05Sm	Li_6_La_2.95_Sm_0.05_Zr_0.5_Nb_0.5_Ta_0.5_Hf_0.5_O_12_	∼94	cubic garnet, LaAlO_3_ La_2_Li_0.5_Al_0.5_O_4_	2.7	highest σ, but multiphase
0.03Sm	Li_6_La_2.97_Sm_0.03_Zr_0.5_Nb_0.5_Ta_0.5_Hf_0.5_O_12_	∼91	cubic garnet, trace unidentified phases	2.3	near phase-pure, σ ≈ undoped
0.01Sm	Li_6_La_2.99_Sm_0.01_Zr_0.5_Nb_0.5_Ta_0.5_Hf_0.5_O_12_	∼90	single cubic garnet	1.6	phase-pure, σ ≈ undoped

The Sr-substituted sample (Li_6_La_2.95_Sr_0.05_Zr_0.5_Nb_0.5_Ta_0.5_Hf_0.5_O_12_) shows a single-phase cubic garnet structure, and no secondary reflections were detected within the limits of XRD. This suggests that Sr^2+^ is incorporated into the La^3+^ site without noticeably destabilizing the lattice. The ionic size mismatch between Sr^2+^ (1.260 Å, CN = 8) and La^3+^ (1.160 Å, CN = 8) is relatively small (∼8.6%), which falls within the range usually tolerated in the garnet framework. Charge compensation is most likely related to Li vacancy formation, described by reaction Sr + Li + La_La_^×^+V_Li_^′^ = La + Sr_La_^′^+Li_Li_^×^.

By contrast, Ba^2+^ and Sm^3+^ substitutions give rise to additional phases. In the Ba-doped sample, extra reflections were observed and matched to Ba_2_LaZrO_5.5_ (PDF 48-0334, purple arrows), a layered perovskite-type phase. This is consistent with the larger ionic radius of Ba^2+^ (1.420 Å, CN = 8), which is about 22% greater than that of La^3+^ and exceeds the typical tolerance for garnet substitution. The large mismatch likely introduces local strain and promotes segregation during sintering. For Sm^3+^ substitution, reflections corresponding to LaAlO_3_ (PDF 31-0022, green arrows) and La_2_Li_0.5_Al_0.5_O_4_ (PDF 40-1167, black arrows) were identified. Although Sm^3+^ (1.079 Å, CN = 8) is closer in size to La^3+^ (−7.0%), these secondary phases may arise from limited Sm solubility in the garnet lattice, possibly combined with minor Al contamination from the crucible during synthesis.

Taken together, these results suggest that the incorporation of dopant in HE garnet (LLZNTH) is still constrained by both size and chemistry. Even though configurational entropy can help stabilize multication systems, local tolerance limits remain important for phase stability. In this study, the case of Sr^2+^ shows that a moderate size mismatch (around 10%) combined with simple charge compensation is sufficient to achieve homogeneous substitution. This may serve as a practical guideline when considering future dopants for garnet-type electrolytes.

Among the different La-site dopants examined in Figure 1, the Sm-substituted sample exhibited the highest ionic conductivity (see the section below for details), suggesting a beneficial role of Sm incorporation in enhancing Li-ion transport. However, at the 0.05 substitution level, XRD analysis revealed the presence of secondary phases, indicating that excessive Sm addition exceeds the solubility limit of the garnet lattice. To reconcile the improved conductivity with structural stability, a series of LLZNTH-Sm_*x*_ samples (*x* = 0.01, 0.03, 0.05) was synthesized to systematically examine the influence of Sm content on phase purity and establish the solubility limit, as shown in Figure 2. It is worth noting that although the Ba-doped sample also shows a slight improvement in ionic conductivity, the large ionic radius of Ba^2+^ leads to pronounced phase segregation and limits further enhancement. Therefore, only the Sm-doping series was selected for a more detailed compositional investigation.

**Figure 2:**
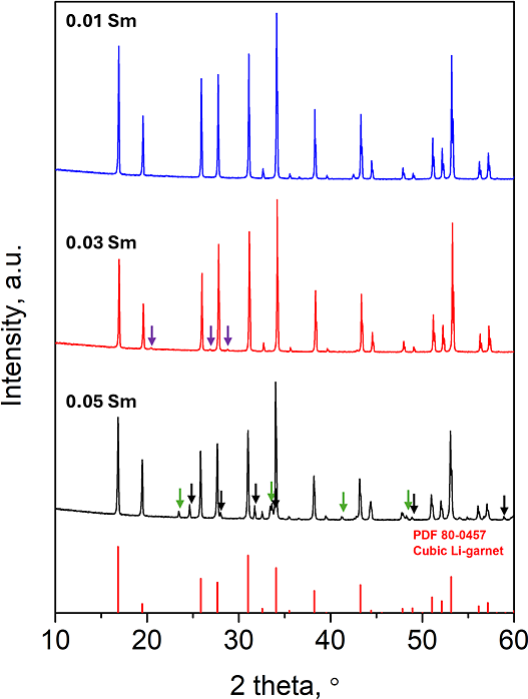
XRD patterns of LLZNTH samples with different Sm substitution contents (0.01, 0.03, and 0.05). All samples retain the cubic garnet phase (PDF 80-0457). For the 0.05 Sm sample, secondary reflections corresponding to LaAlO_3_ (PDF 31-0022) and La_2_Li_0.5_Al_0.5_O_4_ (PDF 40-1167) are observed.

Reducing the Sm content to 0.03 leads to substantial suppression of the secondary phases observed at 0.05 substitution. While the major secondary reflections corresponding to LaAlO_3_ and La_2_Li_0.5_Al_0.5_O_4_ are eliminated, a few weak, unidentified reflections remain (marked with purple arrows). These minor impurity peaks, which could not be indexed to known phases in the database, suggest that 0.03 Sm approaches the solubility limit of Sm^3+^ in the LLZNTH garnet structure. The significant reduction in secondary phase intensity indicates that this composition represents a near-critical doping level where the garnet framework can accommodate most, but not all, of the Sm^3+^ dopants homogeneously. At the lowest substitution level (*x* = 0.01), the XRD pattern exhibits a phase-pure cubic garnet structure with no detectable secondary phases within the detection limits of the XRD analysis. The clean diffraction pattern demonstrates successful homogeneous incorporation of Sm^3+^ into the La^3+^ sublattice without inducing competing crystallization pathways. This composition clearly falls within the solubility limit of the garnet framework, indicating that Sm^3+^ can be accommodated through the charge compensation mechanism without exceeding the structural tolerance of the high-entropy garnet matrix. The systematic phase evolution from 0.05 to 0.01 Sm establishes that the solubility limit of Sm^3+^ in the LLZNTH garnet lies between 0.01 and 0.03 mole fractions, with 0.03 representing a near-critical composition. This relatively low solubility limit, compared to conventional LLZO systems, may reflect the already complex cation environment in the high-entropy base composition (Li_6_La_3_Zr_0.5_Nb_0.5_Ta_0.5_Hf_0.5_O_12_), which could reduce the available configurational space for additional Sm^3+^ incorporation.

These results demonstrate that even in high-entropy systems, where configurational entropy can extend solid solution ranges, fundamental structural constraints still govern dopant solubility. The successful achievement of a single-phase garnet at 0.01 Sm provides a composition that maintains structural integrity while introducing controlled lattice perturbation through Sm^3+^ substitution. However, the critical question remains whether this optimized phase purity translates to enhanced ionic conductivity or whether the structural disorder present in the multiphase 0.05 composition plays a beneficial role in Li^+^ transport.

The microstructures and elemental distributions of LLZNTH and La-site-doped samples sintered at 1150 °C for 4 h were examined by SEM and EDS (Figure 3; some summaries are also shown in Table 1). Clear differences in grain growth, porosity, and elemental uniformity were observed, and these trends generally match the phase behavior seen in the XRD.

**Figure 3:**
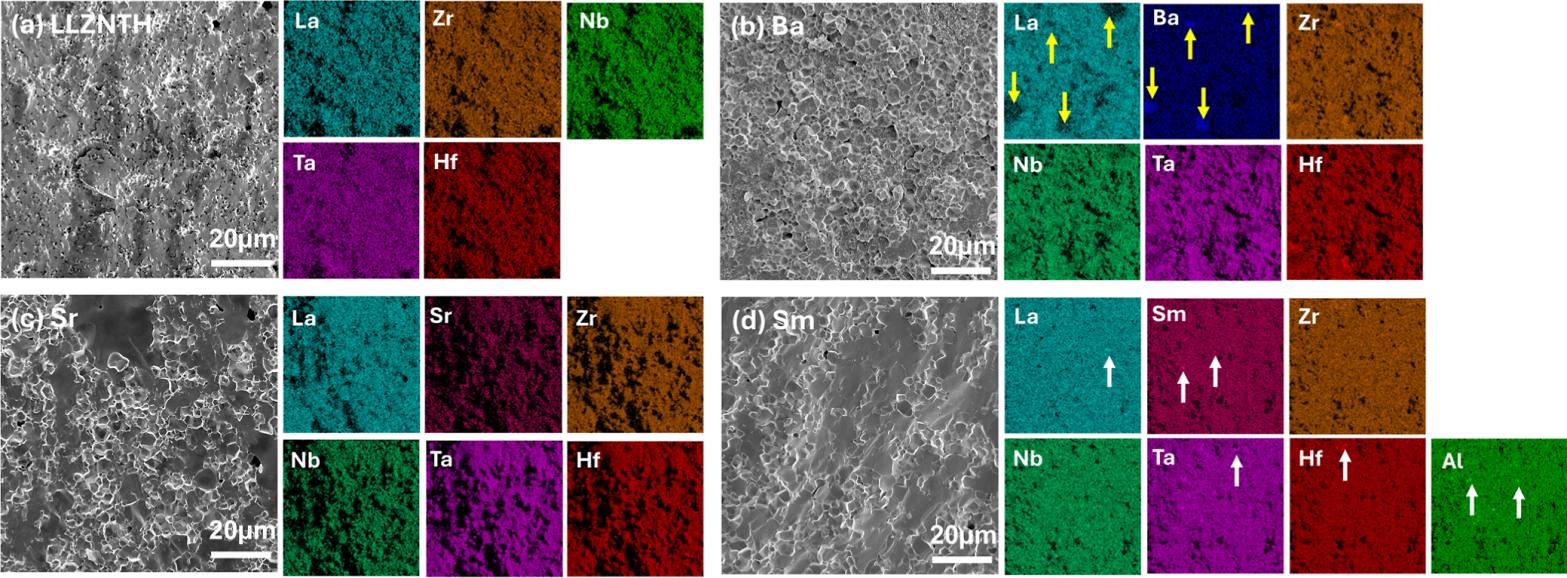
SEM micrographs and EDS mapping of the sintered samples. (a) LLZNTH nondoped, (b) Ba-doped, (c) Sr-doped, and (d) Sm-doped.

The undoped LLZNTH sample (Figure 3a) shows a fine-grained structure (∼2–5 μm) with substantial porosity. The relative density is ∼89%, suggesting incomplete densification under the chosen conditions. This is consistent with the known difficulty of sintering high-entropy garnets without additives. Per our previous research, sintering at 1200 °C for 4 h can lead to a much higher density (∼95%).^14,26^ For comparison purposes, we set the temperature at 1150 °C for 4 h for this research to evaluate the effects of different La site dopants.

Ba substitution (Figure 3b) produces much larger grains (∼10–15 μm) and reduced porosity (relative density–91%). However, EDS mapping reveals Ba- and La-rich areas that coincide with each other, while Zr, Nb, Ta, and Hf remain evenly distributed. This segregation pattern is in line with the secondary Ba_2_LaZrO_5.5_ phase identified by XRD. The Sr-doped sample (Figure 3c) shows intermediate grain sizes (∼5–10 μm) but the highest density (∼95%). Importantly, the EDS maps indicate that Sr and the other cations are homogeneously distributed, and no segregation features were found. This uniform elemental distribution provides direct microstructural evidence for the successful single-phase garnet formation observed in XRD analysis, confirming that Sr^2+^ can be accommodated homogeneously within the La^3+^ sublattice without inducing phase separation. In contrast, the Sm-doped sample (Figure 3d) displays a more irregular grain morphology and evidence of intergranular fracture. The porosity is lower than in undoped LLZNTH, but EDS reveals clear Sm enrichment in certain regions. Ta, Hf, La, and Al also appear uniformly distributed, while local Al enrichment is observed at some grain boundaries. These features correlate with the LaAlO_3_ and La_2_Li_0.5_Al_0.5_O_4_ phases detected by XRD and suggest a limited solubility of Sm in the garnet framework.

All La-site substitutions improve densification relative to undoped LLZNTH, following the order Sr (95%) > Sm (94%) > Ba (91%) > LLZNTH (89%). This enhancement can be attributed to the flux-like behavior of the dopant oxides, which promote mass transport during sintering. However, the relationship between densification and phase purity is complex: while Sr achieves both optimal density and single-phase formation, Ba and Sm show improved densification accompanied by phase separation, indicating that enhanced sintering kinetics do not guarantee phase stability.

The microstructural analysis indicates that successful La-site doping in LLZNTH requires balancing multiple factors: (1) ionic size compatibility for homogeneous substitution, (2) thermodynamic stability to prevent phase separation, and (3) sintering behavior for adequate densification. Sr doping achieves this balance optimally, combining a homogeneous elemental distribution with excellent densification. In contrast, Ba and Sm doping improves densification but sacrifices phase homogeneity, creating complex microstructures that may influence ionic transport through multiple pathways including bulk conduction, interfacial transport, and secondary phase contributions.

Electrochemical impedance spectroscopy (EIS) was used to evaluate the ionic transport properties of LLZNTH and La-site-substituted samples (Figure 4). The Nyquist plots show the expected semicircles for Li-garnet electrolytes, and the data were fitted with equivalent circuits to extract bulk and grain boundary contributions. The undoped LLZNTH sample (Figure 4a) has the lowest conductivity (1.7 × 10^–4^ S cm^–1^). The large semicircle diameter reflects high overall resistance, consistent with its fine grains (2–5 μm) and high porosity (relative density–89%). The broad semicircle suggests that grain boundary resistance dominates the response. Doping improves the conductivity in all cases. Ba doping (Figure 4b) gives only a modest increase (1.9 × 10^–4^ S cm^–1^). Although grain growth and densification are evident, the Ba_2_LaZrO_5.5_ phase and Ba–La segregation likely add extra grain boundary resistance, limiting the benefit. The Sr-substituted sample (Figure 4c) reaches 2.3 × 10^–4^ S cm^–1^ and remains phase-pure with a uniform elemental distribution, which agrees with the XRD and SEM results. Sm substitution shows a different behavior. At a 0.05 content (Figure 4d), the conductivity is the highest (2.7 × 10^–4^ S cm^–1^) even though several secondary phases are present. Previous research on Li_0.625_Al_0.125_H_0.25_Cl_0.75_O_0.25_ also reports the formation of secondary phases, which, rather than reducing, actually enhance the total ionic conductivity.^40^ However, to further clarify the mechanisms underlying the enhanced ionic conduction, detailed insights from NPD analysis are discussed in a later section. The smaller semicircle indicates reduced resistance, suggesting that lattice distortion or interfacial effects may contribute to Li^+^ transport. In contrast, lower Sm levels (0.01 and 0.03) give conductivities close to the undoped sample, despite higher phase purity. This trend points to a threshold effect: only when the Sm content is high enough to significantly perturb the lattice does the conductivity increase, though this comes at the cost of secondary phases.

**Figure 4:**
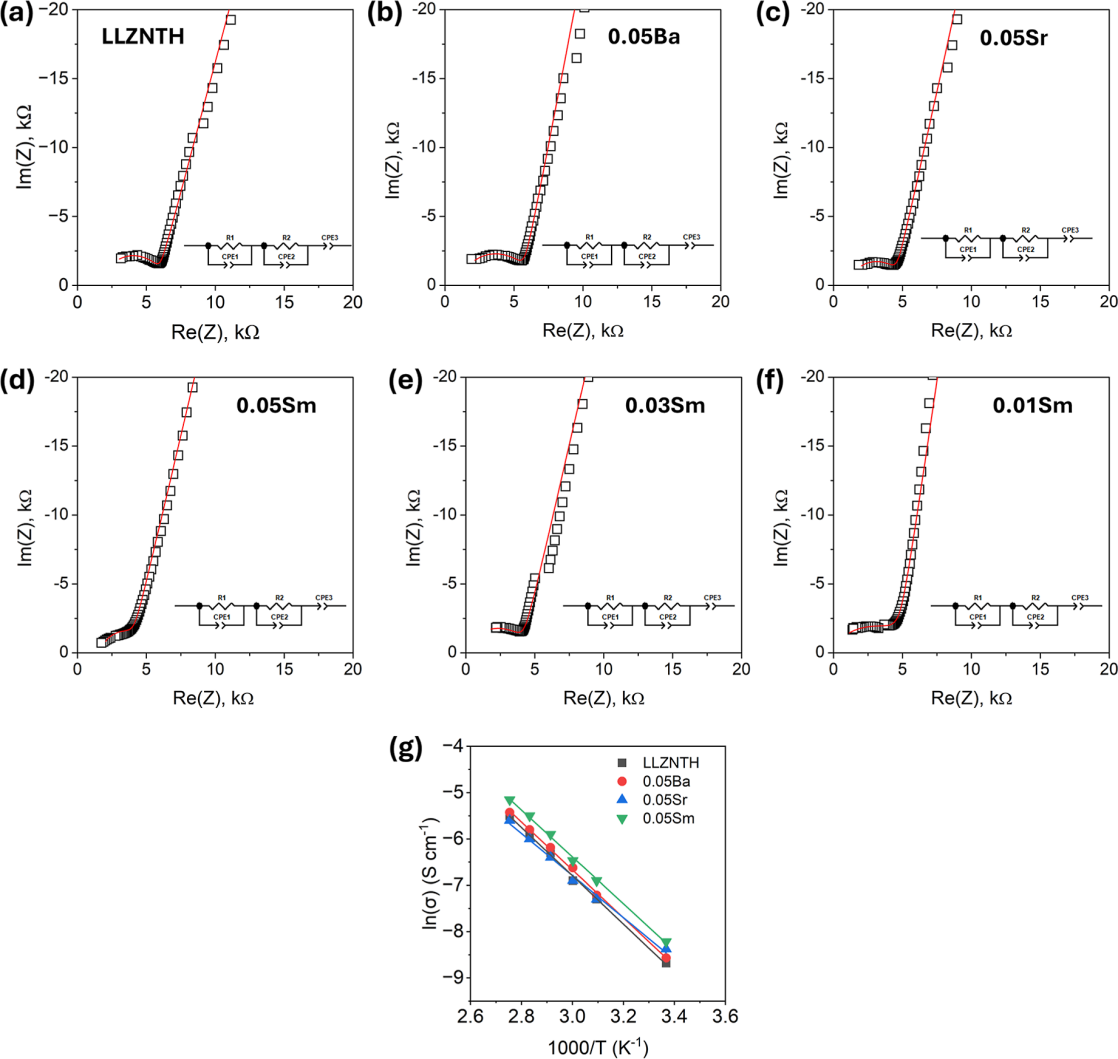
Electrochemical impedance spectra of sintered LLZNTH samples at 1150 °C for 4 h: (a) undoped LLZNTH, (b) Ba-doped, (c) Sr-doped, (d) Sm_0.05_-doped, (e) Sm_0.03_-doped, (f) Sm_0.01_-doped, and (g) activation energy. Experimental data are shown as open symbols, and fitted curves are plotted as red solid lines using equivalent circuit models (insets).

Overall, the data suggest that conductivity in these high-entropy garnets is influenced by more than just the phase purity. As a summary, there are multiple factors that determine the conductivity: (1) improved densification reducing porosity-related resistance, (2) optimized charge compensation creating Li^+^ vacancies, (3) lattice distortion effects modifying Li^+^ migration barriers, and (4) potential interfacial transport contributions from secondary phases. The high conductivity of Sm_0.05_ indicates that the latter two factors can outweigh the detrimental effects of phase separation when the dopant content exceeds solubility limits. Such results further demonstrate that HE Li-garnet optimization requires balancing multiple competing factors rather than simply maximizing phase purity. While Sr doping provides a single phase and high relative density, the Sm_0.05_ results demonstrate that controlled structural disorder can yield superior performance, albeit with increased complexity.

Arrhenius analysis (Figure 4g) shows that all compositions exhibit activation energies for Li^+^ transport close to literature numbers but with a clear distinction between the undoped and doped samples. The pristine LLZNTH composition has the highest activation energy of ∼0.451 eV, consistent with its lower room-temperature ionic conductivity (1.7 × 10^–4^ S cm^–1^). Upon introduction of Ba, Sr, or Sm dopants, the activation energies decrease markedly and fall within a narrow, tightly grouped range of ∼0.434–0.438 eV. Ba substitution reduces the barrier to ∼0.438 eV, accompanied by a slight conductivity enhancement (1.9 × 10^–4^ S cm^–1^). Sr doping yields the lowest Ea (∼0.434 eV) among the single-phase garnets, and its conductivity increases more significantly (2.3 × 10^–4^ S cm^–1^), aided by improved densification (∼95%). Sm-doped LLZNTH exhibits an activation energy comparable to Sr (∼0.436 eV) yet attains the highest conductivity (2.7 × 10^–4^ S cm^–1^), suggesting that Li redistribution into 96 h sites and the entropy-stabilized local environment help offset the influence of secondary phases such as LaAlO_3_ and La_2_Li_0.5_Al_0.5_O_4_. These results demonstrate a consistent trend: doping significantly lowers the intrinsic migration barrier relative to the undoped garnet, but the activation energies of the three doped compositions are similar, indicating that their different dopant chemistries tune the local Li-ion transport landscape in subtly distinct yet energetically comparable ways.

Neutron powder diffraction (NPD) and Rietveld refinement were carried out to investigate the effect of La-site doping on the crystal structure and Li-ion distribution in the LLZNTH, as shown in Figure 5 and summarized in Table 2. All samples crystallize in the cubic garnet structure with space group *Ia*3̅*d*, with refined lattice parameters in the range of 12.9023–12.9111 Å. The refinement quality factors (Rwp = 3.09–4.28% and χ^2^ = 2.982–4.807) confirm the reliability of the structural models.

**Figure 5:**
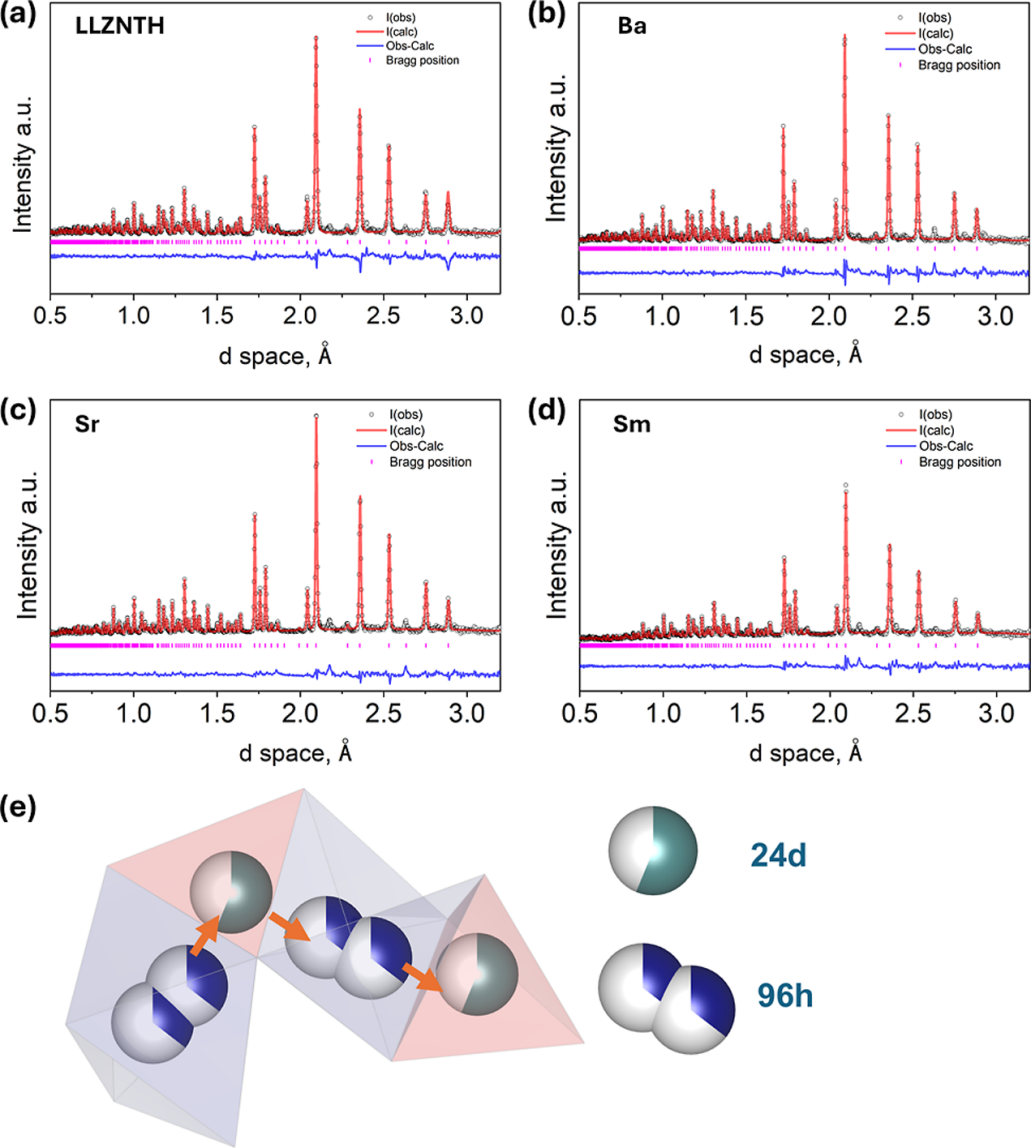
NPD and the associated Rietveld refinement to reveal the crystal structures of the samples. (a) LLZNTH nondoped, (b) 0.05Ba-doped, (c) 0.05Sr-doped, and (d) 0.05Sm-doped. (e) The typical Li-ion migration pathways in LLZO and the role of 96 h occupancy on the total ionic conductivity.

**Table 2:** Crystallographic Parameters of LLZNTH, Ba, Sr, and Sm Doped Samples Based on the Refinement of NPD Data

	R_wp_ (%)	χ^2^	lattice parameter (Å)	site	occupancy
LLZNTH Li_6_La_3_Zr_0.5_Nb_0.5_Ta_0.5_Hf_0.5_O_12_	4.28	4.807	12.9024	Li1 (24 d)	0.5993
				Li2 (96 h)	0.350
Ba Li_6_La_2.95_Ba_0.05_Zr_0.5_Nb_0.5_Ta_0.5_Hf_0.5_O_12_	4.00	4.306	12.9023	Li1 (24 d)	0.603
				Li2 (96 h)	0.354
Sr Li_6_La_2.95_Sr_0.05_Zr_0.5_Nb_0.5_Ta_0.5_Hf_0.5_O_12_	3.09	2.982	12.9111	Li1 (24 d)	0.648
				Li2 (96 h)	0.342
Sm Li_6_La_2.95_Sm_0.05_Zr_0.5_Nb_0.5_Ta_0.5_Hf_0.5_O_12_	3.56	3.364	12.9099	Li1 (24 d)	0.571
				Li2 (96 h)	0.357

A key observation from the refinement is the redistribution of Li between the tetrahedral 24 d (Li1) and octahedral 96 h (Li2) sites. Upon doping, the Li distribution shifts noticeably. In the Sr-doped sample, the Li2 occupancy decreases slightly to 0.342, whereas Ba- and Sm-doping results in increased Li2 occupancies of 0.354 and 0.357, respectively. This higher Li2 (96 h) occupancy is particularly significant as the 96 h sites constitute the high-mobility positions that form the continuous three-dimensional Li-ion conduction network. The enhanced population of these sites is considered the primary factor contributing to improvements in total ionic conductivity.^32,41^

The Li-site occupancy visualization (Figure 5e) reveals the profound impact of this redistribution on the ionic transport pathways. In undoped LLZNTH, the low 96 h occupancy (0.350) creates a fragmented conduction network with limited pathway connectivity, forcing Li^+^ ions to rely on less efficient hopping mechanisms. The Sm-doped sample shows enhanced 96 h site occupancy/population, creating a well-connected three-dimensional Li^+^ conduction network that facilitates long-range ionic transport through continuous hopping pathways. This Li-site redistribution provides the fundamental mechanistic explanation for the conductivity trends observed in the EIS analysis. The increase in 96 h occupancy in Sm-doped samples translates to enhanced Li^+^ mobility through several mechanisms: (1) increased availability of high-mobility sites, (2) improved pathway connectivity reducing bottlenecks, (3) optimized hopping distances between adjacent 96 h sites, and (4) enhanced percolation of the three-dimensional conduction network.

The differential effects of La-site dopants on the Li distribution can be attributed to their distinct local structural influences. Sr^2+^ and Ba^2+^, despite different sizes, both maintain relatively conventional Li distributions, suggesting limited perturbation of the Li sublattice. Sm^3+^, however, appears to create local structural distortions or charge distribution effects that preferentially stabilize Li^+^ in 96 h sites, possibly through modified Li–O bonding environments or altered electrostatic potential landscapes. These findings refine our understanding of conductivity enhancement in high-entropy garnets, demonstrating that Li-site engineering through strategic dopant selection can be more impactful than conventional approaches focused solely on phase purity or lattice parameter optimization. The ability of Sm to drive Li redistribution while maintaining an acceptable structural integrity (despite secondary phases) suggests that controlled local disorder can be beneficial for ionic transport applications.

The NPD provides direct evidence that La-site doping in HE LLZNTH can alter Li-site occupancy, which consequently affects total conductivity. The discovery that Sm promotes 96 h site population indicates the counterintuitive observation that multiphase Sm-doped samples outperform single-phase alternatives, potentially challenging conventional design paradigms in SSE development.

To further evaluate the practical applicability of these garnet electrolytes in real battery environments, the electrochemical stability and critical current density (CCD) of Li|garnet|Li symmetric cells were subsequently examined. As shown in Figure 6, the CCD of the symmetric cells is 0.40 mA cm^–2^ for the undoped and Ba-doped samples, 0.25 mA cm^–2^ for the Sr-doped sample, and 0.20 mA cm^–2^ for the Sm-doped sample. All samples exhibit stable Li plating/stripping at low current densities, confirming good initial interfacial contact with lithium metal. However, the observed CCD variation reveals a delicate balance between ionic conductivity and mechanical/interfacial robustness. Although the Sm-doped sample shows the highest total ionic conductivity, its lower CCD suggests that enhanced bulk transport alone does not guarantee interfacial stability. The presence of minor secondary phases and local lattice distortion introduced by Sm^3+^ substitution may soften grain boundaries and reduce the critical current threshold before short-circuiting occurs. In contrast, the Ba-doped and undoped samples, despite their lower conductivities, exhibit higher CCD values, likely due to their more uniform microstructures and denser grain packing, which provide stronger mechanical resistance to dendrite penetration. A similar report also indicated that codoping Ta and Ba into LLZO improved the CCD.^35^ The Sr-doped sample lies between these two extremes, consistent with its intermediate conductivity. It should be noted that no direct correlation between the CCD and total ionic conductivity was observed. CCD is a complex parameter influenced by multiple factors including ionic and electronic transport, grain size, relative density, secondary phases, and interfacial properties. Therefore, this cannot be explained by bulk conductivity alone. Nonetheless, the CCD results provide valuable insight into how La-site doping collectively affects the electrochemical stability and interfacial robustness of high-entropy garnet electrolytes.^8,42^

**Figure 6:**
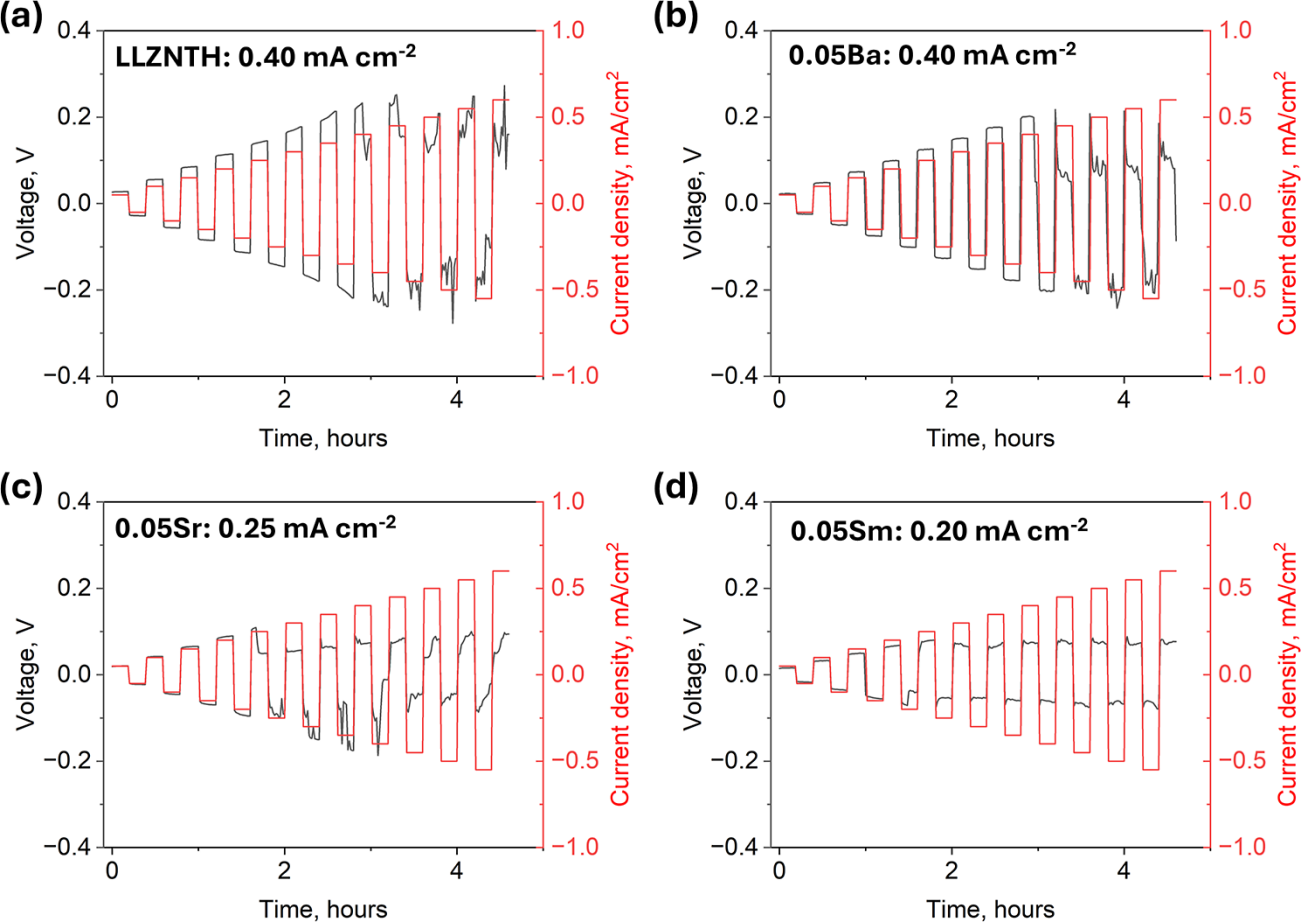
Critical current density (CCD) measurements of Li|garnet|Li symmetric cells for (a) undoped LLZNTH, (b) 0.05Ba-doped, (c) 0.05Sr-doped, and (d) 0.05Sm-doped samples. The initial current density was 0.05 mA cm^–2^, and the step increment was 0.05 mA cm^–2^ per cycle. The voltage profiles were recorded to evaluate the electrochemical stability and Li stripping/plating behavior of each electrolyte.

It should be noted that all samples eventually experienced soft shorting behavior rather than sudden hard failure, implying that the interfacial instability is gradual and likely governed by mechanical deformation and Li filament growth within the porous grain boundaries. Similar La-site substitution effects on CCD have also been reported in recent studies, where dopants with mismatched ionic radii or charge states were found to alter interfacial stress distribution and Li nucleation behavior. These results collectively indicate that La-site doping influences not only the bulk Li^+^ transport but also the microstructural integrity governing interfacial stability. Optimizing both densification and Li-site distribution is, therefore, critical to simultaneously achieve high conductivity and high CCD in high-entropy garnet electrolytes.

From the above electrochemical results, including both ionic conductivity and CCD measurements, it can be concluded that although the configurational entropy increase from La-site substitution is relatively small compared to that from the Zr-site multication framework, the induced local lattice distortion and site disorder remain decisive in governing Li^+^ distribution and transport behavior. This highlights the multiscale nature of entropy–structure–property coupling in garnet-type electrolytes. Overall, this work provides a compositional design principle for balancing phase stability, conductivity, and interfacial integrity in high-entropy garnet electrolytes, thereby offering guidance for the future development of robust solid-state electrolytes for practical Li–metal batteries.

## 3 Conclusions

The present study establishes clear structure–property correlations in La-site-doped high-entropy garnets (LLZNTH: Li_6_La_3_Zr_0.5_Nb_0.5_Ta_0.5_Hf_0.5_O_12_):

- Sr substitution stabilizes a phase-pure garnet with homogeneous cation distribution, yielding moderate conductivity enhancement.
- Ba substitution promotes grain growth and densification but induces Ba-containing secondary phases, limiting ionic transport improvement.
- Sm substitution (0.05) delivers the highest ionic conductivity (2.7 × 10^–4^ S cm^–1^), despite multiphase formation, highlighting a trade-off between conductivity gain and phase stability.
- Lower Sm contents (0.01–0.03) suppress secondary phases but fail to enhance conductivity, indicating that lattice perturbation is essential for transport improvement.
- La-site dopants uniformly reduce the activation energy for Li-ion transport when compared with the undoped LLZNTH.
- Neutron powder diffraction refinements reveal that Sm incorporation redistributes Li into the high mobility 96 h sites, enhancing the connectivity of Li^+^ migration pathways.

Overall, La-site dopants not only influence the phase stability and microstructure but also directly tune Li sublattice occupancy. This provides a design principle for optimizing ionic conductivity in high-entropy garnet electrolytes by balancing dopant solubility, phase stability, and Li-site redistribution.

## 4 Experiments

### 4.1 Sample Fabrications

Following the chemical formulas listed in Table 1, the powders were prepared through a solid-state synthesis method.^26^ Stoichiometric amounts of LiOH·H_2_O with 10% excess (Li is easy to evaporate during synthesis and sintering; therefore, excess amounts were added, 98% minimum purity, Thermo Scientific), La_2_O_3_ (99.99% purity, Thermo Scientific, dried at 950 °C), ZrO_2_ (99.9% purity, Inframat Advanced Materials), Nb_2_O_5_ (99.9% purity, Alfa Aesar), Ta_2_O_5_ (99.99% purity, Inframat Advanced Materials), HfO_2_ (99%, Thermo Scientific), BaCO_3_ (99.9%, Alfa Aesar), SrCO_3_ (99.9%, Alfa Aesar), and Sm2O3 were ball milled for 1 h using a zirconia milling tank and balls and isopropyl alcohol (IPA, >99.5%, VWR) as the liquid media on a shaker mill (SPEX 8000, SPEX SamplePrep). The mixture was dried at 105 °C, ground, and pressed into pellets. Calcination occurred in an alumina crucible at 900 °C for 12 h in air. Subsequent 2 h of ball milling using zirconia milling media in IPA was performed on the shaker mill to reduce the particle size. The milled powders were uniaxially pressed into pellets under a pressure of ∼60 MPa. The green pellets were surrounded by the same powder and placed in an alumina crucible with a lid to minimize the evaporation of Li during sintering. The pellets were sintered at 1150 for a duration of 4 h to achieve the highest ionic conductivity for each sample. The heating and cooling rates were 3 °C/min. The reported properties in this paper are summarized in Table 1.

### 4.2 Sample Characterizations

Phases of the samples were characterized by X-ray diffraction (XRD, Empyrean, Malvern Panalytical). The density of the sintered sample was determined through Archimedes’ principle using IPA as the immersion liquid, and the relative density was calculated correspondingly. Microstructures and element distributions of the sintered pellets were characterized by a scanning electron microscope (Apreo 5, Thermo Scientific) equipped with energy-dispersive X-ray spectroscopy (EDS). The samples were also characterized by neutron powder diffraction (NPD) performed on VULCAN at Oak Ridge National Laboratory.^43^ The neutron diffraction patterns were refined through the GSAS software package to obtain crystallographic structural parameters.^44^

To determine the ionic conductivity, Li-ion blocking silver paste was applied on the polished samples and fired at 700 °C for 0.5 h to ensure a good interface. The ionic conductivity of each sample was determined by electrochemical impedance spectroscopy (EIS, SP-300, BioLogic) in a frequency range of 7 MHz to 1 Hz using the potentiostatic EIS model with a 100 mV amplitude at room temperature (∼20 °C). The results were fitted by using appropriate equivalent circuit models to obtain ionic conductivity. The conductivity was also determined at elevated temperatures up to 90 °C to obtain the activation energy.

To determine the critical current density (CCD), Li-garnet-Li symmetric cells were assembled. Sintered pellets were polished to a thickness of ∼400 μm by using SiC sandpapers with IPA as the lubricant. The polished pellets were then heated at 400 °C to remove surface impurities, following literature procedures.^45^ The treated pellets were subsequently pressed into clean molten Li metal at ∼250 °C inside an Ar-filled glovebox to ensure intimate interfacial contact between cells.^46^ The initial current density was set to 0.05 mA cm^–2^ and increased in increments of 0.05 mA cm^–2^.
